# The Use of Peripheral Blood-Derived Stem Cells for Cartilage Repair and Regeneration *In Vivo*: A Review

**DOI:** 10.3389/fphar.2020.00404

**Published:** 2020-04-03

**Authors:** You-Rong Chen, Xin Yan, Fu-Zhen Yuan, Jing Ye, Bing-Bing Xu, Zhu-Xing Zhou, Zi-Mu Mao, Jian Guan, Yi-Fan Song, Ze-Wen Sun, Xin-Jie Wang, Ze-Yi Chen, Ding-Yu Wang, Bao-Shi Fan, Meng Yang, Shi-Tang Song, Dong Jiang, Jia-Kuo Yu

**Affiliations:** ^1^Knee Surgery Department of the Institute of Sports Medicine, Peking University Third Hospital, Beijing, China; ^2^School of Clinical Medicine, Weifang Medical University, Weifang, China

**Keywords:** peripheral blood, stem cell, cartilage, *in vivo*, review

## Abstract

**Background:**

Peripheral blood (PB) is a potential source of chondrogenic progenitor cells that can be used for cartilage repair and regeneration. However, the cell types, isolation and implantation methods, seeding dosage, ultimate therapeutic effect, and *in vivo* safety remain unclear.

**Methods:**

PubMed, Embase, and the Web of Science databases were systematically searched for relevant reports published from January 1990 to December 2019. Original articles that used PB as a source of stem cells to repair cartilage *in vivo* were selected for analysis.

**Results:**

A total of 18 studies were included. Eight human studies used autologous nonculture-expanded PB-derived stem cells (PBSCs) as seed cells with the blood cell separation isolation method, and 10 animal studies used autologous, allogenic or xenogeneic culture-expanded PB-derived mesenchymal stem cells (PB-MSCs), or nonculture-expanded PBSCs as seed cells. Four human and three animal studies surgically implanted cells, while the remaining studies implanted cells by single or repeated intra-articular injections. 121 of 130 patients (in 8 human clinical studies), and 230 of 278 animals (in 6 veterinary clinical studies) using PBSCs for cartilage repair achieved significant clinical improvement. All reviewed articles indicated that using PB as a source of seed cells enhances cartilage repair *in vivo* without serious adverse events.

**Conclusion:**

Autologous nonculture-expanded PBSCs are currently the most commonly used cells among all stem cell types derived from PB. Allogeneic, autologous, and xenogeneic PB-MSCs are more widely used in animal studies and are potential seed cell types for future applications. Improving the mobilization and purification technology, and shortening the culture cycle of culture-expanded PB-MSCs will obviously promote the researchers' interest. The use of PBSCs for cartilage repair and regeneration *in vivo* are safe. PBSCs considerably warrant further investigations due to their superiority and safety in clinical settings and positive effects despite limited evidence in humans.

## Introduction

Articular cartilage covering the surface of joints plays a very important role in bearing loads, absorbing mechanical shocks, and enabling synovial joints to articulate with low friction (Chen et al., 2017). Acute trauma, repetitive joint use, and degenerative joint disease may lead to cartilage and osteochondral injuries (Saw et al., 2011; Fu et al., 2014a). Articular cartilage has a very limited regenerative and self-healing potential due to its avascular, aneural, and alymphatic characteristics and a low number of progenitor cells (Redondo et al., 2018). Many attempts have been made to identify the ideal treatment for cartilage lesions, including bone marrow stimulation (BMS) techniques (Jin et al., 2011), osteochondral autografts and allografts (Makris et al., 2015), and cell-based cartilage repair procedures, including autologous chondrocyte implantation (ACI) (Riboh et al., 2017), mesenchymal stem cell (MSC)-based therapy (Fu et al., 2014a; Li et al., 2016) and tissue-engineered cartilaginous grafts (Zhao et al., 2018; Ding et al., 2019; Wang et al., 2019; Zhang et al., 2019). Since BMS techniques, osteochondral transplantation, and ACI have limitations and shortcomings, such as fibrocartilage regeneration, donor site complications, graft failure, dedifferentiation of seed cells, and two-stage invasive surgical procedures (Fortier et al., 2010; Andriolo et al., 2017; Riboh et al., 2017), MSCs, which are multipotent progenitor cells with an intrinsic potential for multilineage differentiation, self-renewal, low immunogenicity, anti-inflammatory activity, and immunomodulatory effects by suppressing the graft-versus-host reaction, may be obtained from multiple tissues of individual patients, and these cells are easily cultured, amplified, and purified (Goldberg et al., 2017; Guadix et al., 2017). MSCs are widely used in cartilage repair and regeneration as seed cells without concerns regarding increasing the risk of cancer (Hernigou et al., 2013; Liu et al., 2018; Han et al., 2019). An increasing number of studies have suggested that peripheral blood (PB) is a potential alternative source of MSCs, which have shown similar chondrogenic differentiation potential with bone marrow-derived MSCs (BM-MSCs) in both *in vitro* and *in vivo* studies (Fu et al., 2014a; Wang et al., 2016a). PB-derived stem cells (PBSCs) can be obtained by a minimally invasive procedure with fewer complications than bone marrow (BM) harvesting, which has been reportedly associated with haemorrhage, chronic pain, neurovascular injury, and even death (Bain, 2003). Moreover, PBSCs also have the ability to be used in autologous transplantation, which greatly benefits patients in clinical applications and facilitates the development of a one-stage surgical solution and other cell-based therapies (Spaas et al., 2012; Hopper et al., 2015a; Saw et al., 2015).

Although increasing evidence has shown that PBSCs are a potential alternative source of chondrogenic progenitor cells for cartilage repair, reviews describing the application of PBSCs for cartilage repair and regeneration *in vivo* are lacked. The purpose of this review was to evaluate the treatment efficacy and safety of using PBSCs for cartilage regeneration *in vivo* and attempt to clarify treatment details about cell types, isolation methods, optimal dosages, and implantation methods.

## Method

This review was conducted in accordance with Preferred Reporting Items for Systematic Reviews and Meta-Analyses (PRISMA) guidelines and a PRISMA checklist using PubMed, EMBASE, and Web of Science to search for relevant studies published from 1 January 1990 to 31 December 2019 (Charlesworth et al., 2019). The search terms used in the selection were “(peripheral OR blood OR circulating OR circulation) AND (mesenchymal OR stem cell OR stromal cell OR progenitor cell OR mononuclear cell OR primitive cell) AND (cartilage OR chondrogenesis OR chondral OR osteochondral OR osteoarthritis) AND (vivo OR human OR patient OR animal OR mouse OR rat OR rabbit OR dog OR sheep OR pig OR horse OR ovine)”.

YRC, XY, and FZY independently screened study titles and abstracts from the beginning. Only original research studies published in full English that used PB as the source of chondrogenic progenitor cells for cartilage repair were included in the analysis. Both print journals and e-published journals were eligible for inclusion and screening. However, all non-English language studies, review articles, letters, editorials, conference, patents, and meeting abstracts and studies not involving cartilage regeneration were excluded. Duplicates were excluded. In addition, studies of primary cells that were not derived from the PB and studies that were not related to *in vivo* animal or human experiments or only used non-PB sources were excluded. Disagreements between the authors were resolved by discussion and consensus.

**Figure 1:**
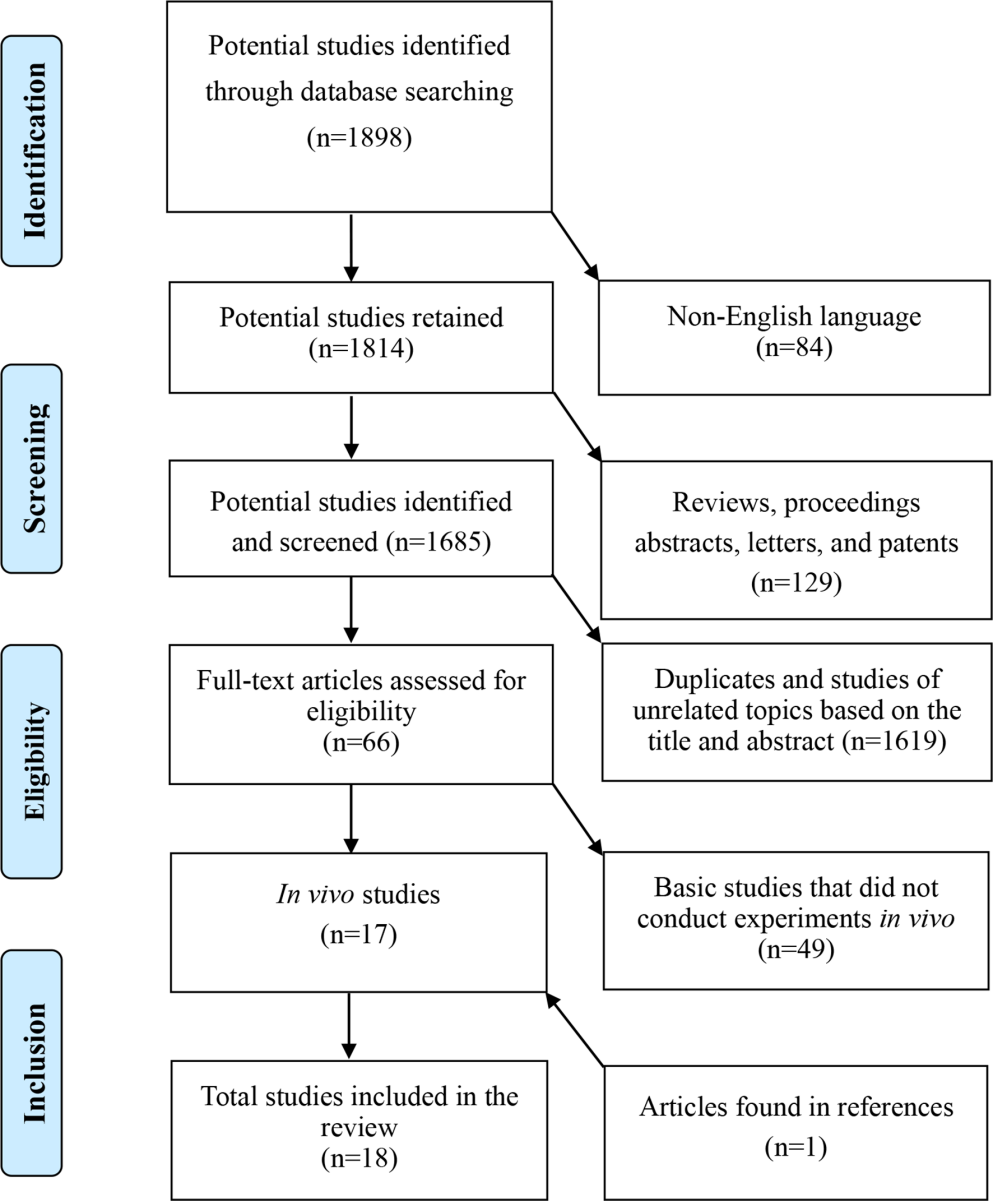
A flowchart of the literature search.

To avoid the omission of relevant studies, we investigated all reference lists of the eligible studies for studies that were likely not identified by the initial retrieval criteria. Unpublished studies were not included in this review. A flowchart of the literature search is shown in **Figure 1**. We reviewed human studies first, and then reviewed the animal studies according to the order of the publication date. Preoperative characteristics of patients and animals, treatment details, and the treatment efficacy and safety of PBSCs were assessed.

## Results

### Description of the Included Studies

Overall, 1,898 publications were retrieved from the initial search. A total of 1,685 potential studies were retained for further identification after 84 non-English language studies and 129 review articles, letters, editorials, conference, and meeting abstracts were excluded. Furthermore, 1,619 duplicates and studies of unrelated topics based on the title and abstract, and 49 basic studies that did not conduct experiments *in vivo* were excluded. We identified 17 *in vivo* studies consisting of 7 human trials and 10 animal studies published between 1990 and 2019 using this retrieval strategy. All reference lists of the 17 included studies were investigated, and an additional human trial (Jancewicz et al., 2004) was identified and included in this review. Finally, data from the 18 studies [8 human studies (Jancewicz et al., 2004; Saw et al., 2011; Skowroński et al., 2012; Saw et al., 2013; Skowroński and Rutka, 2013; Turajane et al., 2013; Fu et al., 2014a; Saw et al., 2015) and 10 animal studies (Spaas et al., 2012; Broeckx et al., 2014a; Broeckx et al., 2014b; Fu et al., 2014a; Deng et al., 2015; Hopper et al., 2015b; Zhao et al., 2018; Daems et al., 2019; Broeckx et al., 2019a; Broeckx et al., 2019b) published by investigators from seven countries or regions] were analyzed.

Among the 18 studies, 7 were case reports [6 in humans (Jancewicz et al., 2004; Saw et al., 2011; Skowroński et al., 2012; Turajane et al., 2013; Fu et al., 2014a; Saw et al., 2015) and 1 in horses (Spaas et al., 2012)], 1 was a human comparative study (Skowroński and Rutka, 2013), 1 was a human randomized controlled trial (RCT) (Saw et al., 2013), 1 was a preliminary study (in horses) (Broeckx et al., 2014a), 1 was a pilot study (in horses) (Broeckx et al., 2014b), 4 involved animal models [rabbits (Fu et al., 2014b), sheep (Hopper et al., 2015b), rats (Deng et al., 2015), and pigs (Zhao et al., 2018), 1 was a prospective placebo-controlled study (in dog) (Daems et al., 2019), and 2 were randomized, double-blinded, placebo-controlled proof-of-concept study (in horses) (Broeckx et al., 2019a; Broeckx et al., 2019b) (**Table 1**).

**Table 1:** Preoperative characteristics of patients and animals.

	Study ID	Species (no. of subjects)	Study design	The age/weight of patients or animals	Clinical or imaging examination	Types of defects or diseases	Location of lesions	The defect size/ICRS grade
**Human studies**	(Jancewicz et al., 2004)	Human (9)	Case report	N/A	N/A	Osteochondral defects	Talus	0.5×0.7 cm with 0.5-1.0 cm depth ICRS IV
	(Skowroński et al., 2012)	Human (52)	Case report	16-55 years old	N/A	Cartilage lesions	Patella (22), medial femoral condyle (38), lateral femoral condyle (6)	4 to 12 cm^2^ (mean 6.2 cm^2^), ICRS grade III and IV
	(Skowroński and Rutka, 2013)	Human (46)	Comparative study	7-52 years old (average age: 26 years)	N/A	Osteochondral lesions	Medial femoral condyle	>4 cm^2^, > 6 mm deep, ICRS grade IV
	(Turajane et al., 2013)	Human (5)	Case report	52-59 years old (median age: 56 years)	Varus deformity (1.20 ± 0.84°); Kellegan Lawrence stages 1-3	Early-stage OA	Medial condyle (4), patellofemoral (1)	ICRS grade III and IV
	(Saw et al., 2011)	Human (5)	Case report	19-52 years old	N/A	Chondral defects	Knee	ICRS grade III and IV
	(Saw et al., 2013)	Human (50)	RCT	22-50 years old	N/A	Chondral defects	Knee	ICRS grade III and IV
	(Saw et al., 2015)	Human (8)	Case report	50-56 years old	Varus deformity	End-stage OA (bone-on-bone lesions)	Medial compartment of the knee joint	ICRS grade IV
	(Fu et al., 2014a)	Human (1)	Case report	19 years old	N/A	Full-thickness cartilage defects	Lateral femoral trochlea	4 cm^2^ ICRS grade IV
**Animal studies**	(Spaas et al., 2012)	Horse (1)	Case report	5 years old	Severe unilateral forelimb lameness; new periarticular bone formation	Degenerative joint disease	Pastern joint	N/A
	(Broeckx et al., 2014a)	Horse (50)	Preliminary study	N/A	Mild to moderate clinical lameness; positive flexion test	Degenerative joint disease	Fetlock joint	N/A
	(Broeckx et al., 2014b)	Horse (165)	Pilot study	N/A	Clinical lameness; locomotory disorder; positive flexion test	Degenerative joint disease	Stifle joint (30), fetlock joint (58), coffin joint (43), pastern joint (34)	N/A
	(Fu et al., 2014b)	New Zealand white rabbit (30)	Animal models	4 months old	N/A	Full-thickness osteochondral defects	Trochlear groove of the distal femur	5 mm in diameter and 1-2 mm in depth
	(Hopper et al., 2015b)	Mountain sheep (24)	Animal models	3-5 years old	N/A	Full-thickness osteochondral defects	Medial femoral condyle (MFC)	6.0 mm in diameter and 8 mm in depth
	(Deng et al., 2015)	SD rat (28)	Animal models	12 weeks old	N/A	Papain-induced OA model	Knee joints	N/A
	(Zhao et al., 2018)	Diannan small-ear pig (12)	Animal models	N/A (average weight: 15 kg)	N/A	Chondral defects	Medial and lateral femoral condyles (MFC and LFC)	7 mm in diameter and 4 mm in depth
	(Broeckx et al., 2019a)	Horse (12)	A randomized, double-blind, placebo-controlled proof-of-concept study	Median age: 8.5 years old	Mild cartilage changes and normal synovium (arthroscopic examination)	Surgically induced osteoarthritis	The right fetlock joint	Mild cartilage changes (superficial wear line, partial erosion, minor irregularity or thinner cartilage spots)
	(Broeckx et al., 2019b)	Horse (75)	A randomized, multicenter, double-blinded, and placebo-controlled study	3-23 years old	Grade 2 or 3 lameness on the AAEP scale; mild-to-moderate response to flexion test; mild-to-moderate joint swelling	Degenerative joint disease	Fetlock joint	Early staged fetlock degenerative joint disease lasting for at least 2 months
	(Daems et al., 2019)	Dog (6)	A prospective placebo-controlled study	5-10 years old	Stable pain and lameness lasting for over 1 month	OA	Humeroradial joint	Mild to severe OA

N/A, not available; ICRS, International Cartilage Repair Society; RCT, randomized controlled trial; OA, osteoarthritis; AAEP, American Association of Equine Practitioners.

### Preoperative Characteristics of the Patients and Animals

The age of the patients ranged from 7 to 59 years in the 8 human studies (Jancewicz et al., 2004; Saw et al., 2011; Skowroński et al., 2012; Saw et al., 2013; Skowroński and Rutka, 2013; Turajane et al., 2013; Fu et al., 2014a; Saw et al., 2015). Lesions were mainly located in the tibial plateaus (Saw et al., 2011; Saw et al., 2013), patella (Saw et al., 2011; Skowroński et al., 2012; Saw et al., 2013), femoral condyles (Saw et al., 2011; Skowroński et al., 2012; Saw et al., 2013; Skowroński and Rutka, 2013; Turajane et al., 2013; Saw et al., 2015), femoral trochlea (Saw et al., 2011; Saw et al., 2013; Turajane et al., 2013; Fu et al., 2014a), intercondylar notch (Saw et al., 2011), and talus of the ankle joint (Jancewicz et al., 2004). The types of lesions included cartilage defects (Saw et al., 2011; Skowroński et al., 2012; Saw et al., 2013; Fu et al., 2014a), osteochondral defects (Jancewicz et al., 2004; Skowroński and Rutka, 2013), and early- and late-stage osteoarthritis (Turajane et al., 2013; Saw et al., 2015). The International Cartilage Repair Society (ICRS) scores were all grade III–IV (Jancewicz et al., 2004; Saw et al., 2011; Skowroński et al., 2012; Saw et al., 2013; Skowroński and Rutka, 2013; Turajane et al., 2013; Fu et al., 2014a; Saw et al., 2015).

Types of lesions included spontaneous and induced osteoarthritis (Spaas et al., 2012; Broeckx et al., 2014a; Broeckx et al., 2014b; Deng et al., 2015; Daems et al., 2019; Broeckx et al., 2019a; Broeckx et al., 2019b), cartilage defects (Zhao et al., 2018), and osteochondral defects (Fu et al., 2014b; Hopper et al., 2015b) in the 10 animal studies. The lesions were in the knee joint (Broeckx et al., 2014a; Fu et al., 2014b; Deng et al., 2015; Hopper et al., 2015b; Zhao et al., 2018), fetlock joint (Broeckx et al., 2014a; Broeckx et al., 2014b; Broeckx et al., 2019a; Broeckx et al., 2019b), pastern joint (Broeckx et al., 2014a), coffin joint (Broeckx et al., 2014b), and humeroradial joint (Daems et al., 2019). The preoperative characteristics of the patients and animals, such as the age, clinical and imaging examination, types of defects and diseases, location of lesions, and defect size/ICRS grade are shown in **Table 1**.

### Stem Cell Types and Isolation Methods

Eight human studies (Jancewicz et al., 2004; Saw et al., 2011; Skowroński et al., 2012; Saw et al., 2013; Skowroński and Rutka, 2013; Turajane et al., 2013; Fu et al., 2014a; Saw et al., 2015) and 1 animal (Hopper et al., 2015b) study used autologous nonculture-expanded condensed PBSCs, 1 animal study (Deng et al., 2015) used allogenic condensed PBSCs, 1 animal study (Spaas et al., 2012) used autologous culture-expanded PB-MSCs, 6 animal studies (Broeckx et al., 2014a; Broeckx et al., 2014b; Fu et al., 2014b; Zhao et al., 2018; Broeckx et al., 2019a; Broeckx et al., 2019b) used allogenic culture-expanded PB-MSCs, and 1 animal study (Daems et al., 2019) used xenogeneic culture-expanded PB-MSCs as seed cells for cartilage repair and regeneration.

All 8 human studies (Jancewicz et al., 2004; Saw et al., 2011; Skowroński et al., 2012; Saw et al., 2013; Skowroński and Rutka, 2013; Turajane et al., 2013; Fu et al., 2014a; Saw et al., 2015) with 130 patients used a blood cell separator to collect PBSCs. One animal study (Deng et al., 2015) with 28 Sprague-Dawley (SD) rats used the density gradient centrifugation (DGC) method to isolate PBSCs. Eight animal studies (Spaas et al., 2012; Broeckx et al., 2014a; Broeckx et al., 2014b; Hopper et al., 2015b; Zhao et al., 2018; Daems et al., 2019; Broeckx et al., 2019a; Broeckx et al., 2019b) with 272 horses, 24 mountain sheep, 12 Diannan small-ear pigs, and 6 dogs used the DGC and plastic adherence (PA) methods to isolate PB-MSCs and PBSCs. Furthermore, one animal study (Fu et al., 2014b) with 30 New Zealand White rabbits used the erythrocyte lysis and PA methods to isolate PB-MSCs.

### Cell density, Dosage, and Implantation Methods

The seeding dosage in 5 human studies (Saw et al., 2011; Saw et al., 2013; Skowroński and Rutka, 2013; Fu et al., 2014a; Saw et al., 2015) and 1 animal study (Deng et al., 2015) using nonculture-expanded PBSCs as seed cells ranged from 5.0×10^6^ to 3.5×10^7^ cells/ml (or cells/injection), and the seeding dosage in 2 human studies (Skowroński et al., 2012; Turajane et al., 2013) and 1 animal study (Hopper et al., 2015b) was less than 5.0×10^6^ cells/ml (or cells/injection). In 5 animal studies using PB-MSCs as seed cells, the seeding dosage in 3 studies ranged from 1×10^6^ to 5.0×10^6^ cells/ml (or cells/injection) (Spaas et al., 2012; Fu et al., 2014b; Daems et al., 2019; Broeckx et al., 2019a; Broeckx et al., 2019b). One human study (Jancewicz et al., 2004) and 3 animal studies(Broeckx et al., 2014a; Broeckx et al., 2014b; Zhao et al., 2018) did not mention the cell seeding dosage.

Four human studies (Jancewicz et al., 2004; Skowroński et al., 2012; Skowroński and Rutka, 2013; Fu et al., 2014a) and 3 animal studies (Fu et al., 2014b; Hopper et al., 2015b; Zhao et al., 2018) implanted cells by surgery, while the remaining 4 human studies (Saw et al., 2011; Saw et al., 2013; Turajane et al., 2013; Saw et al., 2015) and 7 animal studies (Spaas et al., 2012; Broeckx et al., 2014a; Broeckx et al., 2014b; Deng et al., 2015; Daems et al., 2019; Broeckx et al., 2019a; Broeckx et al., 2019b) implanted cells by single or repeated intra-articular injections.

### Other Therapies and Postoperative Rehabilitation

All human studies used a variety of other treatments, such as intra-articular debridement (Jancewicz et al., 2004; Saw et al., 2011; Skowroński et al., 2012; Saw et al., 2013; Skowroński and Rutka, 2013; Turajane et al., 2013; Fu et al., 2014a; Saw et al., 2015), the modified sandwich technique (Jancewicz et al., 2004; Skowroński and Rutka, 2013), BMS (Saw et al., 2011; Skowroński et al., 2012; Saw et al., 2013; Saw et al., 2015), high tibial osteotomy (HTO) (Saw et al., 2011; Saw et al., 2015), and patellofemoral realignment (Fu et al., 2014a), to promote cartilage repair and regeneration while implanting cells. Strict rehabilitation programmes and passive or active exercises (Saw et al., 2011; Saw et al., 2013; Skowroński and Rutka, 2013; Turajane et al., 2013; Fu et al., 2014a; Saw et al., 2015) were followed to avoid early weight bearing, joint stiffness, and adhesion.

The animal studies used other treatments, such as decalcified bone matrix (DBM) scaffolds (Fu et al., 2014a; Zhao et al., 2018), collagen-glycosaminoglycan (GAG) scaffolds (Hopper et al., 2015b), platelet-rich plasma (PRP) injections (Broeckx et al., 2014a; Broeckx et al., 2014b), and equine allogeneic plasma (EAP) (Broeckx et al., 2019a; Broeckx et al., 2019b), while implanting cells. Except for 2 studies (Broeckx et al., 2019a; Broeckx et al., 2019b), there were no strict rehabilitation plans in the other animal studies.

**Table 2** summarizes the details of the application of PBSCs to cartilage repair and regeneration in humans and animals.

**Table 2:** Treatment Details of PBSCs for Cartilage Repair and Regeneration in Humans and Animals.

	Study ID	Cell types	Cell sources and blood volume (ml)	Isolation methods	Cellular constituent characterization	Cell dose	Cell stage (passage number)	Method of delivery	Surgical procedures	Rehabilitation
**Human studies**	(Jancewicz et al., 2004)	PBSCs	Autologous G-CSF-activated PB	Blood cell separation	CD34^+^	N/A	Fresh condensed stem cells (P0)	Surgical implantation	Debridement+sandwich technique	N/A
	(Skowroński et al., 2012)	PBSCs	Autologous G-CSF-activated PB, 40-80 ml	Blood cell separation	N/A	8×10^5^ - 3.2×10^6^ cells/ml	Fresh condensed stem cells (P0)	Surgical implantation	Debridement + BMS+ PBSC suspension with collagen membrane cover+treatment of co-existing pathologies	N/A
	(Skowroński and Rutka, 2013)	PBSCs	Autologous G-CSF-activated PB, 40 ml	Blood cell separation	N/A	1.25×10^6^ - 5.2×10^6^ cells/ml	Fresh condensed stem cells (P0)	Surgical implantation	Debridement+modified sandwich technique	Passive and active exercises, non-weight to full-weight bearing
	(Turajane et al., 2013)	PBSCs	Autologous hG-CSF-activated PB	Leukapheresis	CD34^+^: 0.34% to 1.04%; CD105^+^: 0.75% to 0.88%; chondrogenic differentiation	TNC: 2.67- 5.99×10^3^ cells/injection	Fresh or cryopreserved condensed stem cells (P0)	Repeated IA injections (3 times)	Debridement +BMS+repeated IA injections (PBSCs+ GFAP +hG-CSF +HA)	Non-weight bearing (ambulation with axillary crutch)
	(Saw et al., 2011)	PBSCs	Autologous G-CSF-mobilized PB	Apheresis	(i) Fresh PBSCs: CD34^+^: 1.86%; CD105^+^: 7.24%; (ii) Frozen PBSCs: CD34^+^: 1.22%; CD105^+^: 8.39%	2.0×10^7^ cells/injection (CD105^+^cells)	Fresh or cryopreserved condensed progenitor cells (P0)	Repeated IA injections (2 times)	Debridement +BMS+HTO(1)+repeated IA injections	CPM+ crutch-assisted partial to full weight bearing
	(Saw et al., 2013)	PBSCs	Autologous G-CSF-mobilized PB	Apheresis	(i) Fresh PBSCs: CD34^+^: 1.86%; CD105^+^: 7.24%; (ii) Frozen PBSCs: CD34^+^: 1.22%; CD105^+^: 8.39%	2.0×10^7^ cells/injection (CD105^+^cells)	Fresh or cryopreserved condensed stem cells (P0)	Repeated IA injections (8 times)	Debridement +BMS+repeated IA injections	CPM+ crutch-assisted partial to full weight bearing
	(Saw et al., 2015)	PBSCs	Autologous G-CSF-mobilized PB	Apheresis	(i) Fresh PBSCs: CD34^+^: 1.86%; CD105^+^: 7.24%; (ii) Frozen PBSCs: CD34^+^: 1.22%; CD105^+^: 8.39%	1.0-2.0×10^7^ cells/injection (CD105^+^cells)	Cryopreserved condensed stem cells (P0)	Repeated IA injections (7 times)	Debridement +BMS+HTO+repeated IA injections	CPM, crutch-assisted partial to full weight bearing
	(Fu et al., 2014a)	PBSCs	Autologous rhG-CSF-mobilized PB	Blood cell separation	N/A	3.496×10^7^ cells/ml	Fresh condensed stem cells (P0)	Surgical implantation	Debridement+PBSCs with autologous periosteum flap cover+ patellofemoral realignment	Strict rehabilitation programme
**Animal studies**	(Spaas et al., 2012)	PB-MSCs	Autologous PB, 10 ml	DGC and PA	CD29^+^, CD44^+^, CD90^+^, CD79α^-^, MHC II^-^, trilineage differentiation	2.5×10^6^ cells/injection	Culture-expanded cells (P1, P3)	Repeated IA injections (2 times)	N/A	N/A
	(Broeckx et al., 2014a)	PB-MSCs (native or chondrogenic induction)	Allogeneic PB 50 ml	DGC and PA	(i) Native: CD29^+^, CD44^+^, CD90^+^, CD105^+^; CD45^-^, CD79a^-^, MHC II^-^ and a monocyte/macrophage marker; trilineage differentiation; p63^-^, low in MHC I, Ki67^+^, Col II^+^, and Vimentin^+^. (ii) Chondrogenic induction: aggrecan^+^, Col II^+^, COMP^+^, p63^+^ and GAG^+^; decrease in Ki67.	N/A	Culture-expanded cells (P4)	Single IA injection	PB-MSCs with or without PRP	N/A
	(Broeckx et al., 2014b)	PB-MSCs (native or chondrogenic-induced)	Allogeneic PB 50 ml	DGC and PA	(i) Native: CD29^+^, CD44^+^, CD90^+^, CD105^+^; CD45^-^, CD79a^-^, MHC II^-^ and a monocyte/macrophage marker; trilineage differentiation; p63^-^, low in MHC I, Ki67^+^, Col II^+^, and Vimentin^+^. (ii) Chondrogenic induction: aggrecan^+^, Col II^+^, COMP^+^, p63^+^ and GAG^+^; decrease in Ki67.	N/A	Culture-expanded cells (P4)	Single IA injection	PB-MSCs with PRP	N/A
	(Fu et al., 2014b)	PB-MSCs	Allogeneic G-CSF-/AMD3100-mobilized PB, 10 ml	Erythrocyte lysis and PA	CD44/CD29^+^, CD45/MHC II^-^, trilineage differentiation	4×10^6^ cells/scaffold	Culture-expanded cells (P3)	Surgical implantation	Establishment of animal model + cell-DBM scaffold complex implantation	Free movement
	(Hopper et al., 2015b)	PBSCs	Autologous PB	DGC and PA cultured under hypoxia	Stro-1^+^, CD44^+^, CD90^+^, CD106^+^, CD105^+^, CD146^+^ and CD166^+^; CD34^-^/CD45^-^; trilineage differentiation	2.0×10^5^; cells/scaffold	Fresh concentrated stem cells	Surgical implantation	Establishment of animal model + cell- collagen-GAG scaffold complex implantation	Full weight bearing
	(Deng et al., 2015)	PBSCs	Allogeneic G-CSF-mobilized PB, 200–500 µl	DGC	CD34^+^ cells (2.8%), CD34^−^ cells (97.2%)	5×10^6^ cells/injection	Cryopreserved condensed stem cells (P0)	Single IA injection	Establishment of animal model + single IA injection (PBSCs+HA)	N/A
	(Zhao et al., 2018)	PB-MSCs	Allogeneic G-CSF-/AMD3100-mobilized PB, 20 ml	DGC and PA	CD34^-^/CD45^-^; CD44^+^/CD90^+^	N/A	Culture-expanded cells (P3)	Surgical implantation	Establishment of animal model + cell-DBM - cytokine scaffold complex implantation	N/A
	(Broeckx et al., 2019a)	Chondrogenic-induced PB-MSCs	Allogeneic PB	DGC and PA	Aggrecan^+^, Col II^+^, COMP^+^, p63^+^ and GAG^+^; decrease in Ki67.	2×10^6^ cells/injection	Culture-expanded chondrogenic-induced cells	Single IA injection	PB-MSCs with EAP	Rested in a box for 1 week after surgery and exercised on a treadmill for the remainder of the study period
	(Broeckx et al., 2019b)	Chondrogenic-induced PB-MSCs	Allogeneic PB 50 ml	DGC and PA	Chondrogenic induction: CD29^+^, CD44^+^, CD90^+^, CD45^-^, MHC II^-^, and a 4.4-fold COMP expression	2×10^6^ cells/injection	Culture-expanded chondrogenic-induced cells (P10)	Single IA injection	PB-MSCs with EAP	A strict rehabilitation protocol
	(Daems et al., 2019)	Chondrogenic-induced PB-MSCs	Xenogeneic PB 50 ml	DGC and PA	Chondrogenic induction: CD44^+^, CD90^+^, MHC II^-^, and a 4.4-fold COMP expression	1×10^6^ cells/injection	Culture-expanded chondrogenic-induced cells (P10)	Single IA injection	PB-MSCs only	Subjected to home confinement and leash walking in the first 10 days after treatment

PBSCs, peripheral blood-derived stem cells; G-CSF, granulocyte colony-stimulating factor; PB, peripheral blood; N/A, not available; TNC, total nucleated cells; IA, intra-articular; GFAP, growth factor addition/preservation; hG-CSF, human granulocyte colony stimulating factor; HA, hyaluronic acid; BMS, bone marrow stimulus; HTO, High Tibial Osteotomy; CPM, continuous passive motion; DGC, density gradient centrifugation; PA, plastic adherence; PB-MSCs, peripheral blood mesenchymal stem/stromal cells; PRP, Platelet-rich plasma; MHC, major histocompatibility complex; Col, collagen; COMP, cartilage oligomeric matrix protein; GAG, glycosaminoglycan; DBM, decalcified bone matrix; ciMSCs, chondrogenic induced mesenchymal stem cells; EAP, equine allogeneic plasma.

### Efficacy and Safety of Treatment

We assessed the adverse events and the clinical, radiographic, and histologic results to determine the treatment efficacy and safety (**Table 3**).

**Table 3:** Efficacy and safety of treatment.

	Study ID	Follow-up period	Clinical outcomes	Radiology	Second-look arthroscopy/ gross morphology evaluation	Histological assessment	Adverse effects
**Human studies**	(Jancewicz et al., 2004)	6 months to 3 years	Improved Magee score	MRI: regenerative tissue with same signals as normal cartilage	N/A	N/A	Longer bone healing (1 patient)
	(Skowroński et al., 2012)	6 years	(i) Improved KOOS and Lysholm scales, relief of VAS scale; (ii) Approximately 90% of patients with good results	MRI: defects were refilled with regenerative tissue	N/A	N/A	(i) Intra-articular adhesions (1 patient); (ii) Joint pain with intermittent exudates and movement limitations (1 patient)
	(Skowroński and Rutka, 2013)	5 years	(i) Improved KOOS and Lysholm scales, relief of VAS scale; (ii) 92% of patients with good results	MRI: satisfactory reconstruction of the cartilaginous surface and good regenerative integration	N/A	N/A	None
	(Turajane et al., 2013)	6 months	Improved WOMAC and KOOS scales	N/A	N/A	Succeeded in regenerating articular cartilage	Mild swelling and discomfort
	(Saw et al., 2011)	10-26 months	N/A	X-ray: reappearance of medial articulation (1)	Regenerated articular cartilage with a smooth surface and excellent integration with the surrounding native cartilage	Regenerated full-thickness articular hyaline cartilage	Minimal discomfort from PBSCs harvesting and IA injection
	(Saw et al., 2013)	18 months	No IKDC score difference compared to the control group	Improved MRI morphologic scores	Regenerated articular cartilage with a smooth surface and excellent integration with the surrounding native cartilage	Improved total ICRS II histologic scores	Deep vein thrombosis (1 patient in the control group)
	(Saw et al., 2015)	15-58 months	Restoration of lower limb alignment	X-ray: reappearance of the medial compartment	Smooth regenerated articular cartilage and excellent integration with the surrounding native cartilage	(i) Improved ICRS II scores; (ii) High-quality cartilage regeneration resembling hyaline cartilage	None
	(Fu et al., 2014b)	7.5 years	Improved IKDC 2000 subjective score, Lysholm score and Tegner score	(i) CT: subchondral bone recovery; (ii) MRI: near normal cartilage-like tissue regeneration	Regenerated articular cartilage with a smooth surface, but with a slightly yellowish and shallow morphology	N/A	None
**Animal studies**	(Spaas et al., 2012)	4 months	Improved visual gait and objective pressure plate analysis	X-ray and B-ultrasound: no considerable changes	N/A	N/A	None
	(Broeckx et al., 2014a)	12 months	(i) Improved short- and long-term clinical evolution scores; (ii) Relief from clinical lameness, flexion pain and joint effusion	N/A	N/A	N/A	None
	(Broeckx et al., 2014b)	18 weeks	(i) Improved short- and long-term clinical evolution scores; (ii) Relief from clinical lameness and locomotor disorder	N/A	N/A	N/A	Moderate flare reaction (without long-term effects, 3 horses)
	(Fu et al., 2014b)	24 weeks	N/A	N/A	Cartilage regeneration comparable to BM-MSCs	Improved histological grading scale	N/A
	(Hopper et al., 2015b)	26 weeks	N/A	N/A	Improved ICRS macroscopic scores	Improved modified O'Driscoll score	None
	(Deng et al., 2015)	6 weeks	N/A	N/A	N/A	Decreased cellular necrosis, apoptosis, loss of chondrogenic proteins, and modified Mankin scores	N/A
	(Zhao et al., 2018)	12 weeks	N/A	N/A	Cartilage regeneration similar to normal cartilage tissue	Improved O'Driscoll score	N/A
	(Broeckx et al., 2019a)	11 weeks	Improvements in visual and objective lameness;	No significant radiographic changes	Significantly less wear lines and synovial hyperaemia	(i) A significantly higher GAG concentration in the synovial fluid; (ii) Significantly enhanced Alcian Blue uptake and area % of COMP and Collagen II in the cartilage adjacent to the osteochondral fragment	None
	(Broeckx et al., 2019b)	3 weeks-1 year	(i) Improved short- and long-term clinical evolution scores; (ii) Relief from clinical lameness, flexion pain and joint effusion	N/A	N/A	N/A	Nasal discharge (2 horse in the IVP group and 1 in the CP group)
	(Daems et al., 2019)	12 weeks	Relief from pain and lameness	No significant radiographic changes	N/A	N/A	One of the six dogs had vomiting and/or diarrhea twice (one after placebo treatment and one after MSCs treatment)

The follow-up time of the 8 human trials ranged from 6 months to 7.5 years. The clinical evaluation results of 5 studies showed that Magee score (Jancewicz et al., 2004), KOOS scales (Skowroński et al., 2012; Skowroński and Rutka, 2013; Turajane et al., 2013), Lysholm scales (Skowroński et al., 2012; Skowroński and Rutka, 2013), WOMAC scales (Turajane et al., 2013), IKDC 2000 subjective score (Fu et al., 2014a) or Tegner score (Fu et al., 2014a) were improved, VAS scales (Skowroński et al., 2012; Skowroński and Rutka, 2013) were relieved, and Skowroski et al. (Skowroński et al., 2012; Skowroński and Rutka, 2013) reported 90 and 92% of patients with good results in 2012 and 2013, respectively. One study (Saw et al., 2013) reported that there was no IKDC score difference compared to the control group. One study (Saw et al., 2015) reported lower limb line recovery, and one study (Saw et al., 2011) did not report clinical evaluation results.

Five animal studies (Spaas et al., 2012; Broeckx et al., 2014a; Broeckx et al., 2014b; Broeckx et al., 2019a; Broeckx et al., 2019b) on horses reported improved visual gait, objective pressure plate analysis, short- and long-term clinical evolution scores, and relief of visual and objective lameness, flexion pain, and joint effusion.

Radiological examination, which is a non-invasive examination method, was widely used to evaluate the efficacy of cartilage repair and regeneration. Seven human studies used MRI (Jancewicz et al., 2004; Skowroński et al., 2012; Saw et al., 2013; Skowroński and Rutka, 2013; Fu et al., 2014a), X-ray (Saw et al., 2011; Saw et al., 2015) or CT (Fu et al., 2014a) to evaluate the repair effect and reported improved MRI morphologic scores, regenerative tissue with the same signal as normal cartilage, subchondral bone recovery, or reappearance of the medial compartment. However, radiological examination was rarely used in the animal studies. Three animal studies (Spaas et al., 2012; Daems et al., 2019; Broeckx et al., 2019a) reported no significant radiographic changes.

Four human studies (Saw et al., 2011; Saw et al., 2013; Fu et al., 2014a; Saw et al., 2015) evaluated cartilage repair with the method of second-look arthroscopy and suggested that cartilage regeneration was comparable to BM-MSCs with improved ICRS macroscopic scores, cartilage regeneration similar to normal cartilage tissue, or significantly fewer wear lines and synovial hyperaemia.

Histopathological examination is the gold standard for evaluation. Four human studies (Saw et al., 2011; Saw et al., 2013; Turajane et al., 2013; Saw et al., 2015) reported high-quality cartilage regeneration resembling hyaline cartilage and/or improved total ICRS II histologic scores. Five animal studies (Fu et al., 2014b; Deng et al., 2015; Hopper et al., 2015b; Zhao et al., 2018; Broeckx et al., 2019a) reported an improved histological grading scale, modified O'Driscoll score, modified Mankin scores, O'Driscoll score, or significantly higher Alcian blue uptake.

In eight human studies, the major adverse events included longer bone healing (1 patient) (Jancewicz et al., 2004), intra-articular adhesions (1 patient) (Skowroński et al., 2012), joint pain with intermittent exudates and motion limitation (1 patient) (Skowroński et al., 2012), mild swelling (Turajane et al., 2013), and minimal discomfort during PBSCs harvesting and intra-articular (IA) injection (Saw et al., 2011). No serious adverse events occurred during the isolation and treatment of PBSCs. In one human study (Saw et al., 2013), a case of deep vein thrombosis occurred in the control group. In animal studies, moderate flare reactions (3 in 165 horses) (Broeckx et al., 2014a), nasal discharge (3 in 75 horses) (Broeckx et al., 2019), and vomiting and diarrhea (1 in 6 dogs) (Daems et al., 2019) occurred without long-term effects.

## Discussion

Researchers have conducted investigations of PBSCs in cartilage repair and regeneration because of the advantages of PBSCs and limitations of chondrogenic progenitor cells from other sources, such as bone marrow (Bain, 2003), synovial membranes (Murata et al., 2018), and adipose tissue (Kuroda et al., 2015). Increasing evidence has shown that PB-MSCs have a similar potential for proliferation and trilineage differentiation as BM-MSCs and might be a promising source of seed cells for cartilage repair (Wang et al., 2016b) since Fernández et al. (Fernandez et al., 1997) reported the presence of stromal cells in hG-CSF-mobilized PB from patients with breast cancer for the first time in 1997. However, PBSCs were not used to treat chondral defects and promote cartilage regeneration *in vivo* until 2004, as reported by investigators in Poland (Jancewicz et al., 2004).

For the first time, this review comprehensively evaluated the feasibility, efficacy, and safety of using PBSCs for cartilage repair and regeneration *in vivo* by analyzing the preoperative characteristics, therapeutic details, outcomes, and adverse events reported in currently published literature. This review might provide new insights and strategies for further foundational research and clinical applications of PBSCs.

Autologous nonculture-expanded PBSCs are easy to harvest and manipulate from G-CSF-activated PB without the concerns of disease transmission, immune rejection, and ethical issues (Fu et al., 2014a; Saw et al., 2015). PBSCs are currently the most commonly used cell type for cartilage repair in all stem cell types derived from PB. It has been demonstrated that nonculture-expanded PBSCs comprise haematopoietic stem cells (HSCs), fibrocytes, a population of MSCs/mesenchymal progenitor cells (MSCs/MPCs), white blood cells, platelets, growth factors, and a small percentage of red blood cells (Stroncek et al., 1997; Cesselli et al., 2009). When PBSCs are being cultured, other impure cell types except MSCs/MPCs are not present anymore. To a certain degree, the cell composition of nonculture-expanded PBSCs is similar to that of the bone marrow-derived buffy coat (BMBC), which is separated from bone marrow using a Ficoll gradient centrifugation system. The bone marrow-derived buffy coat has been widely used as a source of MSCs for cartilage repair and regeneration and has achieved good to excellent results (Fortier et al., 2010; Jin et al., 2011). Several possible mechanisms of action of PBSCs might contribute to cartilage repair. Hopper et al. (Hopper et al., 2015a; Hopper et al., 2015c) found that PBSCs stimulate the upregulation of eight genes associated with chondrogenic differentiation of knee infrapatellar fat pad-derived MSCs, increase the total number of MSCs, increase native chondrocyte migration, and accelerate the rate of cell movement. Exogenous MSCs, HSCs, and growth factors in PBSCs initiate cartilage regeneration and augment endogenous MSC recruitment from bone marrow to subchondral drilling sites (Khaldoyanidi, 2008; Onuora, 2015; Saw et al., 2015). Deng et al. (2015) suggested that PBSCs prevent the progression of papain-induced knee OA in a rat model by reducing articular surface fibrillation, irregularity, and erosion, and by inhibiting chondrocyte necrosis and loss of chondrogenic proteins. HSCs and non-HSCs, such as MSCs, endothelial progenitor cells, and very small embryonic-like (VSEL) cells, contained in PBSCs might play an important role through a paracrine mechanism (Kucia et al., 2007; Onuora, 2015). Although the term “PBSCs” had different expressions in different studies, such as PB progenitor cells (PBPCs) (Saw et al., 2011) and PB mononuclear cells (PBMCs) (Hopper et al., 2015b), we found that the cell acquisition method and cell composition were basically the same. For the convenience of expression, “PBSCs” was used uniformly in this paper.

The transplantation of autologous culture-expanded PB-MSCs requires two procedures for obtaining patient cells and transplanting the cells after cultivation, which prolongs hospital stays, increases costs and risks contamination related to *in vitro* culture, possibly limiting the clinical application of autologous PB-MSCs (Saw et al., 2013; Fu et al., 2014b). Moreover, an age-related decline in MSC numbers, proliferation, and clonogenicity, which lead to more difficult culture *in vitro* and a longer culture cycle than MSCs from other tissue sources, might be another significant cause for the lack of clinical applications of autologous culture-expanded PB-MSCs (Kassis et al., 2006; Bourzac et al., 2010; Chong et al., 2012; Spaas et al., 2013; Wang et al., 2016b). For example, MSCs derived from bone marrow, synovium or adipose tissue reached 80–90% confluence within 7 to 14 days (Zhang et al., 2014; Jin et al., 2016; Shimomura et al., 2016). However, MSCs derived from PB did not achieve the same confluence until about 21 days after primary culture (Chen et al., 2019). It takes longer to obtain the culture-expanded PB-MSCs than other tissue-derived MSCs. The presence of MSCs in human PB is debatable and their identification may be hampered, among others, by: (i) their low frequency in PB of healthy individuals, and (ii) the large biological variations related to donor age, pathology, disease status, and corresponding treatment regimens (Fox et al., 2007; Moll et al., 2019). Most investigators agree that their frequency in blood is low in healthy individuals, but that the amounts of circulating MSCs may increase under special mobilization conditions, thus supporting the notion that MSCs can be transiently found circulating in blood (Moll et al., 2020). Jain et al. provide evidence that MSCs can be found in PB and apheresis product of patients treated with a typical G-CSF-based HSCs mobilization regimen by using flow cytometry (Jain et al., 2020). However, a systematic review strongly indicated the existence of MSCs in the PB of animals (Wang et al., 2016b), this might be because researchers could improve the success rate of PB-MSCs in animal studies by optimizing mobilization and culture procedures, prolonging the culture time, and increasing the number of animals and the frequency of blood drawn (Pitchford et al., 2009; To et al., 2011; Spaas et al., 2013). To et al. noted in a baboon model that MSC mobilization and colony-forming unit fibroblast (CFUF) in PB in response to G-CSF did only occur when adding stem cell factor (To et al., 2011). Pitchford et al. found, that MSCs/CFU-F were not found in mice PB post-mobilization with G-CSF, but when adding vascular endothelial growth factor and CXCR4-antagonist (Pitchford et al., 2009). Spaas et al. systematically studied the isolation and culture methods, cell characteristics, and clinical safety of equine PB-MSCs, and applied them to many veterinary clinical studies, such as promoting cartilage repair, cutaneous wound healing, and healing of tendon and ligament lesions (Spaas et al., 2013; Beerts et al., 2017; Martinello et al., 2018; Broeckx et al., 2019a). Allogenic or xenogeneic MSCs banks, improving the mobilization and purification techniques, and shortening the culture cycle might effectively account for deficiencies in autologous MSCs, reduce the burden on both patients and treatment providers, and promote the development of single-stage procedures (Moroni and Fornasari, 2013; Pescador et al., 2017).

MSCs inhibit immune responses and are not restricted by the HLA system through immune evasion and immune privilege mechanisms (Paterson et al., 2014; Vega et al., 2015). Moreover, the strong immunomodulatory and immunosuppressive properties of MSCs may play an important role in modifying graft-versus-host reactions during allogenic transplantations (Le Blanc and Ringden, 2007). Two animal studies used allogenic native and chondrogenic-induced PB-MSCs as a treatment for degenerative joint disease in horses and significantly improved the short- and long-term effects without serious adverse events (Broeckx et al., 2014a; Broeckx et al., 2014b). Vega et al. (2015) performed an RCT to assess the feasibility and safety of treating osteoarthritis with allogeneic MSCs in humans, and they concluded that allogeneic MSCs might be a convenient and effective alternative to autologous MSCs for the treatment of OA in the knee without serious transplantation-related adverse events. A number of published papers have indicated that transplanted MSCs influence the local microenvironment of cartilage by paracrine actions, such as the secretion of various growth factors, cytokines, and chemokines, to exert anti-inflammatory, anti-apoptotic, and anti-fibrotic effects on chondrocytes(Kuroda et al., 2015; Mancuso et al., 2019). Another possible mechanism of action of MSCs in cartilage repair and regeneration is that transplanted progenitor cells migrate to damaged cartilage areas and differentiate into chondrocytes and osteocytes (Cesselli et al., 2009). The fate of MSCs injected into the articular cavity can be monitored by labelling with green fluorescent protein (GFP) or carboxyfluorescein diacetate succinimidyl ester (CFDA-SE) (Guest et al., 2008; Sato et al., 2012). Murphy et al. found that the implantation of MSCs into the knee joints of goats with OA showed a strong and sustained effect in promoting cartilage repair. However, further tracing the labelled MSCs showed that the cell retention rate was very low, usually about 3%, and most cells disappeared within a few days (Murphy et al., 2003). This suggests that MSCs may not directly differentiate into chondrocytes to participate in tissue repair *in vivo*, but promote cartilage regeneration through other mechanisms. In recent years, more and more researchers believed that exosomes secreted by MSCs played an important role in cartilage repair and regeneration (Marote et al., 2016; Yan and Wu, 2019; Jin et al., 2020; Liu et al., 2020). Exosomes are generally considered as communication vectors between cells, and carry a large number of complex nucleic acids (mRNA and miRNA lncRNA), proteins and lipids that can regulate and restore extracellular matrix (ECM) homeostasis (Colombo et al., 2014). For example, MSCs exosomes with overexpressing of miR-140-5p blocked other Wnt signals *in vitro* by inhibiting v-ral simian leukemia viral oncogene homolog A (RalA) and activating sex determining region Y-box 9 (SOX9), and regulate the expression of Col II and aggrecan (ACAN) *in vivo* to promote cartilage regeneration (Tao et al., 2017). It may also be an important mechanism for PBSCs to promote cartilage repair.

Blood cell separation is the most commonly used method for collecting PBSCs. It is a developed and simple technique that has been widely used in the treatment of systemic blood diseases. In a monocyte suspension isolated by blood cell separation, CD105^+^ cells have been shown to be more abundance than CD34^+^ cells, and the proportion of CD105^+^ cells increased after cryopreservation (Saw et al., 2011). However, there is no study on the subsequent isolation and culture of PB-MSCs from PBSCs collected by blood cell separation. The current standard methods of PB-MSC isolation are DGC (such as Ficoll, Lymphoprep, and Percoll) and PA (Bourzac et al., 2010).

As one of the most fundamental parameters that might influence the outcome of cartilage repair (Gupta et al., 2016), the optimal density or dosage of PBSCs used for cartilage regeneration in different methods and species has not been fully investigated. Skowroński et al. (Skowroński and Rutka, 2013) reported a slightly poorer outcome of cartilage repair in a group treated with a bone marrow concentrate than a group treated with fresh condensed PBSCs, and they attributed this result to the lower cell count in the suspension obtained from bone marrow. The main concern of using nonculture-expanded PBSCs to promote tissue regeneration is the low content of MSCs within harvests. The number of HSCs (with a CD34^+^ surface marker) and MSCs (with a CD105^+^ surface marker) were quantified by flow cytometry in a study carried out by Saw et al. (Saw et al., 2011). The flow cytometry result showed that the proportion of CD105^+^ cells in fresh PBSC suspension was 7.24% (2.32×10^6^ cells/ml). Interestingly, the proportion of CD105^+^ cells reached 8.39% (2.69×10^6^ cells/ml) after cryopreservation. However, the CD105^+^ cell counts vary between different studies. Turajane et al. (Turajane et al., 2013) reported that a proportion of CD105^+^ cells ranging from 0.75 to 0.88%. The difference of the proportion of CD105^+^ cells in the two studies was probably due to the younger patients in the previous study and the older patients in the latter.

To increase the yield of MSCs from autologous PB, repeated intra-articular injections were implemented in some studies (Saw et al., 2011; Saw et al., 2013; Turajane et al., 2013; Saw et al., 2015), and they speculated that this method is more efficacious than a single injection for the enhancement of cartilage repair on the basis of a suggestion from an animal study (Saw et al., 2009). However, repeated IA injections of culture-expanded allogeneic MSCs is not recommended due to a significant adverse response that might be initiated by immune recognition of allogeneic MSCs after a second exposure (Joswig et al., 2017).

Currently, the optimal seeding density of MSCs also remains unknown. A systematic review showed that the dose of MSCs for cartilage repair varies from 2×10^6^–7.7×10^7^ cells in human clinical studies (Goldberg et al., 2017). Gupta et al. (Gupta et al., 2016) found that an MSC dose of 2.5×10^7^ with the IA injection method for treating OA showed the best improvement for relieving pain and the lowest adverse events compared with other higher dose groups. They hypothesized that a higher cell dosage causes cell aggregation and subsequent cell death due to limited space in the knee joint. A prospective RCT demonstrated that an intra-articular injection of cultured MSCs with a mean dose of 1.46×10^7^ cells for treating OA is effective in improving clinical and magnetic resonance observation of cartilage repair tissue (MOCART) scores after a 2-year follow-up (Wong et al., 2013). Given the limited evidence of clinical application of PB-MSCs in cartilage repair and regeneration, the optimal therapeutic dose of PB-MSCs remains to be further studied.

Moreover, a number of studies have reported concomitant procedures, such as abrasion arthroplasty (Beckmann et al., 2015), autologous bone grafting to restore bone mass (Sadlik et al., 2017), treatment of co-existing pathologies (Wong et al., 2013), and BMS (Jin et al., 2011), PRP (Broeckx et al., 2019a) and HA (Charlesworth et al., 2019) to repair cartilage defects. Thus, the abovementioned methods are recommended to supplement PBSCs for cartilage repair and regeneration. A rigorous postoperative rehabilitation programme is required to protect grafts and avoid the effusion of PBSC suspensions (Skowroński and Rutka, 2013; Fu et al., 2014a).

Compared with other tissue-derived MSCs, the culture of PB-MSCs was relatively difficult, which resulted in less reports of its application *in vivo*, but it does not affect its application prospects. On the contrary, it is ethically more suitable for clinical application due to its unique advantages, such as minimally invasive sample acquisition procedure, repeatable sampling, and high recognition of patients (Fu et al., 2014a; Fu et al., 2014b; Wang et al., 2016a; Chen et al., 2019). In this review, we have summarized all the currently published researches on the use of PBSCs for cartilage repair and regeneration *in vivo*. Although only 5 human and veterinary clinical studies (Saw et al., 2013; Skowroński and Rutka, 2013; Daems et al., 2019; Broeckx et al., 2019a; Broeckx et al., 2019b) had a control group, the results were still very useful for readers, and can reflect the progress and problems in this field to a certain extent.

## Conclusion

This review evaluated the use of PBSCs in cartilage repair and regeneration *in vivo* for the first time. Autologous PBSCs are easy to obtain and are free of transmittable diseases, infection risks, and medical ethical restrictions. They are currently the most commonly used cell type for cartilage repair among all stem cell types derived from PB. Blood cell separation technology is developed, simple, and convenient, making it the most commonly used method to obtain PBSC suspensions. Allogeneic culture-expanded PB-MSCs are more widely used in animal research and are potential seed cell types for cartilage repair and regeneration in the future. DGC and PA are the most commonly used methods for PB-MSC isolation. Improving the purification technology and shortening the culture cycle of culture-expanded PB-MSCs will obviously promote the researchers' interest. PBSCs are safe in cartilage repair and regeneration. Although all reviewed articles indicated that using PB as a cell source enhances cartilage repair and regeneration *in vivo* by the IA injection and surgery implantation methods, we should maintain a prudent attitude towards the positive therapeutic effect of PBSCs considering the deficiency of studies with a high level of evidence, incomplete assessment system of outcomes, and combined use of multiple other treatments. In summary, the use of PBSCs in cartilage repair and regeneration warrants considerable efforts for further investigations due to its superiorities and safety in clinical settings and positive effects despite limited evidence in human.

## Author Contributions

Conception and design: J-KY and DJ. Analysis and interpretation of the data: Y-RC, XY, and F-ZY. Drafting of the article: Y-RC, XY, and F-ZY. Information collection and sorting: JY, B-BX, Z-XZ, Z-MM, and JG. Manuscript editing and proofreading: Y-FS, Z-WS, X-JW, and Z-YC. D-YW, B-SF, MY, and S-TS provided oversight. Critical revision of the article for important intellectual content: J-KY and DJ. All authors read and approved the final manuscript.

## Funding

This work was supported by the National Natural Science Foundation of China (Grant Nos. 51773004, 81630056, 51920105006, 31670982) and National Key Research and Development Program (Grant No. 2016YFC1100704).

## Conflict of Interest

The authors declare that the research was conducted in the absence of any commercial or financial relationships that could be construed as a potential conflict of interest.

The handling editor declared a past co-authorship with one of the authors JK-Y.

## Abbreviations

PBSCs, Peripheral blood-derived stem cells; PB-MSCs, Peripheral blood-derived mesenchymal stem cells; DGC, Density gradient centrifugation; PA, Plastic adherence; BMS, Bone marrow stimulation; ACI, Autologous chondrocyte implantation; BM-MSCs, Bone marrow-derived mesenchymal stem cells; PRISMA, Preferred Reporting Items for Systematic Reviews and Meta-analyses; RCT, Randomized controlled trial; HTO, High tibial osteotomy; DBM, Decalcified bone matrix; ICRS, International Cartilage Repair Society; OA, Osteoarthritis; IA, Intra-articular; hG-CSF, Human granulocyte colony stimulating factor; HA, Hyaluronic acid; CPM, Continuous passive motion; PRP, Platelet-rich plasma; MHC, Major histocompatibility complex; COMP, Cartilage oligomeric matrix protein; GAG, Glycosaminoglycan; MRI, Magnetic Resonance Imaging; CT, Computed Tomography; KOOS, Knee injury and osteoarthritis outcome score; VAS, Visual Analogue Scale; WOMAC, The Western Ontario and McMaster Universities Osteoarthritis Index; IKDC, The International Knee Documentation Committee Knee Uation Form; ECM, extracellular matrix; GFP, green fluorescent protein; CFDA-SE, carboxyfluorescein diacetate succinimidyl ester; RalA, v-ral simian leukemia viral oncogene homolog A; SOX9, sex determining region Y-box 9; ACAN, aggrecan; CFUF, colony-forming unit fibroblast.
