# Commentary: Autologous Peripheral Blood Stem Cells (PBSC) are Safe and Effective in Knee Osteoarthritis

**DOI:** 10.3389/fphar.2021.652738

**Published:** 2021-04-20

**Authors:** Konstantinos I. Papadopoulos, Thana Turajane

**Affiliations:** ^1^ THAI StemLife Co., Ltd, Bangkok, Thailand; ^2^ Department of Orthopedics, Police General Hospital, Bangkok, Thailand

**Keywords:** osteoarthritis, knee, peripheral blood, stem cells, granulocyte colony stimulating factor hG-CSF, leukapheresis, autologous, PBSC

## Introduction

We read with interest the review of Chen et al. “The Use of Peripheral Blood-Derived Stem Cells for Cartilage Repair and Regeneration *In Vivo*: A Review” (Chen et al., 2020). Meta-analyses are a key component of evidence-based health care that by pooling together selected randomized controlled trials (RCTs) calculate an overall estimate or of the effect of the intervention under consideration (Moher and Olkin, 1995). The present review only includes one human RCT.

## Materials and Methods

We believe that our RCT in 60 early osteoarthritis patients (of which 40 were actively treated with autologous hG-CSF activated PBSC and 20 received conventional hyaluronic acid treatment) (Turajane et al., 2017) should have been included. Our RCT showed statistically significant avoidance of total knee arthroplasty, and potent, early, and sustained symptom alleviation. Furthermore, our *in vitro* investigation (Turajane et al., 2014) shed light on the autologous PBSC mechanism of action by confirming chondrogenic differentiation potential for autologous PBSC through potentiated Sox9 transcription resulting in sequential COL-2 and aggrecan mRNA increases that ultimately resulted in histologically confirmed increased proteoglycan and glycosaminoglycan content in newly formed hyaline cartilage.

## Discussion

We thus believe that the results presented in the current metanalysis are incomplete since the omitted patient number is half of the included (55 omitted vs. 130 included) (Jancewicz et al., 2004; Saw et al., 2011; Skowroński et al., 2012; Saw et al., 2013; Skowroński and Rutka, 2013; Turajane et al., 2013; Fu et al., 2014a; Saw et al., 2015). Comprising the 2017 RCT of Turajane et al. (Turajane et al., 2017) would have included a second RCT in the review, thus considerably strengthening the conclusion that autologous PBSCs show superiority in procurement, safety, and positive therapeutic effects in clinical settings where cartilage repair and regeneration are required.
